# Oral Health in Extreme Environments: A Global Health Challenge

**DOI:** 10.21142/2523-2754-1403-2026-294

**Published:** 2026-07-10

**Authors:** Claudio Peña-Soto

**Affiliations:** 1 School of Dentistry, Universidad Cientifica del Sur. Lima, Peru cpenas@cientifica.edu.pe. Universidad Científica del Sur School of Dentistry Universidad Cientifica del Sur Lima Peru cpenas@cientifica.edu.pe

Dentistry has traditionally evolved within controlled clinical environments, where prevention, diagnosis, and treatment follow predictable patterns. However, the growing presence of human activity in extreme settings-such as Antarctica, outer space analogs, and other isolated ecosystems-challenges conventional paradigms of oral healthcare. These environments expose individuals to unique combinations of physiological stress, environmental adversity, and behavioral disruption, transforming oral health into a critical yet often overlooked component of overall health and operational performance.

The Antarctic region represents one of the most demanding natural laboratories for studying human adaptation. Characterized by subzero temperatures, low humidity, high ultraviolet radiation, and prolonged isolation, this environment induces complex systemic responses affecting metabolism, immunity, and neuroendocrine regulation. While these systemic effects have been widely documented, oral health has historically received limited attention, despite its well-established connection to systemic conditions and quality of life ^1^^,^^2^.

Recent clinical evidence has begun to bridge this gap. Findings from the ANTAR XXXI expedition, where researchers from the Universidad Cientifica del Sur participated, at the Machu Picchu Antarctic Scientific Station, provide valuable insights into how even short-term exposure to extreme conditions can alter oral clinical markers. After a two-month deployment, participants demonstrated a significant increase in bleeding on probing-an early indicator of gingival inflammation-alongside a clinically relevant decrease in unstimulated salivary flow. Although other parameters such as oral hygiene, salivary pH, and tooth sensitivity did not show statistically significant changes, they exhibited consistent trends toward deterioration, suggesting the onset of functional imbalance in the oral environment ^3^.

These findings are particularly relevant when interpreted within the broader framework of environmental and behavioral stressors. Antarctic expeditions involve substantial changes in daily routines, including disrupted circadian rhythms, altered sleep patterns, increased physical demands, and modified dietary habits. High consumption of carbohydrate-rich foods, frequent snacking, and reliance on caffeinated beverages contribute to a sustained acidic oral environment. Simultaneously, limited access to water, absence of fluoridation, and constraints in hygiene practices exacerbate vulnerability to oral disease ^3^^,^^4^.

Saliva plays a central role in maintaining oral homeostasis, acting as a buffer, antimicrobial medium, and facilitator of tissue repair. The observed reduction in salivary flow under extreme conditions may therefore represent a critical early mechanism linking environmental stress to oral pathology. This is further supported by studies demonstrating that isolation and environmental stress can modulate the oral microbiome and increase pro-inflammatory biomarkers such as interleukins and leptin, reinforcing the concept of oral health as a sensitive indicator of systemic adaptation ^5^.

Importantly, oral health challenges in extreme environments extend beyond biological factors. Logistical constraints play a decisive role. In many Antarctic missions, dental care is not readily available, and medical emergencies are often managed by non-dental personnel with limited equipment. Epidemiological data indicate that dental problems account for a notable proportion of medical incidents in polar expeditions, underscoring the operational implications of inadequate oral health management ^6^. A recent study highlighted the persistent need for dental care among Antarctic personnel, emphasizing that even well-screened individuals remain susceptible to oral complications during the expedition ^7^.

These realities demand a paradigm shift in how oral health is integrated into expedition medicine and global health strategies. First, pre-expedition screening must evolve toward a “zero-risk” model, ensuring that all potential sources of infection or inflammation are addressed prior to deployment. Second, preventive protocols should include the provision of specialized oral care kits, incorporating high-fluoride dentifrices, salivary substitutes, and remineralizing agents tailored to extreme environments. Third, behavioral interventions-such as structured hygiene schedules and nutritional guidance-should be institutionalized as part of mission protocols rather than left to individual discretion.

Moreover, the incorporation of tele-dentistry represents a promising avenue to overcome geographical barriers. Remote diagnostic tools, standardized intraoral imaging, and real-time consultation with specialists can facilitate early detection and management of oral conditions, reducing the risk of complications that may compromise mission success. In this context, oral health should be recognized not merely as a clinical concern but as a determinant of human performance and resilience in extreme settings.

The implications of this emerging evidence extend beyond Antarctica. As humanity continues to explore and inhabit increasingly challenging environments, from deep-sea stations to long-duration space missions, the lessons learned from polar research become directly applicable. Oral health, often perceived as secondary, emerges as a critical component of integrated healthcare systems designed for isolation, autonomy, and sustainability.

In conclusion, extreme environments reveal vulnerabilities in oral health that remain largely invisible in conventional settings. The early alterations observed in periodontal and salivary markers highlight the need for proactive, evidence-based strategies that prioritize prevention, monitoring, and innovation. Dentistry must expand its scope to address these frontiers, embracing its role within global health and human adaptation. Ultimately, safeguarding oral health in extreme conditions is not only a matter of clinical care but a strategic investment in the well-being, performance, and safety of those who venture into the most demanding environments on Earth.
